# Pseudomonas aeruginosa: A Rare Cause of Necrotizing Fasciitis

**DOI:** 10.7759/cureus.106188

**Published:** 2026-03-31

**Authors:** Carrine S Kogulan, Oluwafemi Ajibola

**Affiliations:** 1 Internal Medicine, Kansas City University of Medicine and Biosciences, Joplin, USA; 2 Pulmonary and Critical Care Medicine, Louisiana State University Health Sciences Center, New Orleans, USA

**Keywords:** b cell neoplasm, icu infectious disease, immunocompromised, necrotizing fasciitis, pseudomonas aeruginosa

## Abstract

Necrotizing fasciitis (NF) is a rare but life-threatening soft tissue infection characterized by rapid progression, systemic toxicity, and high mortality. While Group A *Streptococcus* is the most common pathogen, *Pseudomonas aeruginosa* is an uncommon cause, typically reported in immunocompromised patients.

We present the case of a 77-year-old male with chronic lymphocytic leukemia (CLL), chronic kidney disease, and other comorbidities who initially presented to an outside facility with three days of fever and left lower extremity pain. He was admitted with presumed cellulitis, and blood cultures grew *Pseudomonas aeruginosa*. Despite appropriate antibiotic therapy with vancomycin and ceftriaxone, the patient developed worsening pain, septic shock, rhabdomyolysis, and renal failure, prompting transfer to our facility for higher-level care. Examination revealed tense swelling of the left lower extremity, multiple bullae, and severe pain. Given concern for *Pseudomonas* NF, he underwent emergent above-the-knee amputation, followed by multiple debridements. He was subsequently transferred for advanced plastic surgical closure in stable condition.

*Pseudomonas aeruginosa* remains a rare but severe cause of NF, particularly in immunocompromised patients with hematologic malignancies. Immunosuppression likely contributed to the unusual pathogen and aggressive clinical course in this case. Delayed diagnosis appears to be associated with increased mortality, underscoring the importance of timely blood cultures, broad-spectrum empiric coverage, and urgent surgical intervention. This case highlights the critical need for early recognition of *Pseudomonas*-associated NF and consideration of strict guidelines for high-risk populations to improve outcomes.

## Introduction

Necrotizing fasciitis (NF) is a severe, uncommon soft tissue infection associated with rapidly progressive necrosis of fascia, skin, subcutaneous tissue, and muscle. It carries a high mortality rate, largely due to rapid progression and severe sepsis. Group A *Streptococcus* is the most frequently implicated pathogen; however, other aerobic and anaerobic bacteria have also been reported, including *Clostridium perfringens*, *Bacteroides*, *Prevotella*, *Fusobacterium*, *Enterobacteriaceae*, and *Vibrio vulnificus* [1]. Differentiating NF from cellulitis and other soft tissue infections remains a diagnostic challenge, with studies suggesting that only 15-34% of patients receive an accurate initial diagnosis of NF [2]. Immunocompromised individuals, along with those with diabetes and alcohol use disorder, are particularly susceptible [3]. Early recognition combined with prompt surgical intervention is essential for favorable outcomes.

*Pseudomonas aeruginosa* is a gram-negative, rod-shaped bacterium, more commonly associated with pneumonia, wound infections in burn victims, and urinary tract infections related to indwelling catheters. NF due to *Pseudomonas* is rare but can lead to systemic bacteremia with peri-vasculitis [4]. Reported cases of *Pseudomonas*-associated NF most often occur in immunocompromised hosts. Here, we present a rare case of *Pseudomonas aeruginosa* NF in a patient with chronic lymphocytic leukemia (CLL).

## Case presentation

A 77-year-old man with a medical history of chronic kidney disease (CKD), chronic obstructive pulmonary disorder (COPD), hypertension (HTN), hyperlipidemia, untreated CLL, deep vein thrombosis (DVT), and a social history of smoking quarter packs a day, alcohol use around two drinks per week, and daily cannabis use presented to an outside facility for the evaluation of three days history of left lower extremity pain and fever. Physical examination was remarkable for worsening swelling, erythema of the left lower extremity, and significant tenderness to palpation. Laboratory work was remarkable for leukocytosis, azotemia, elevated creatine kinase, and elevated lactic acid (Table 1). Venous Doppler ultrasound of the lower extremity was negative for deep venous thrombosis. The patient was diagnosed with left lower limb cellulitis and started on empiric antibiotics with vancomycin and ceftriaxone. The blood culture grew *Pseudomonas aeruginosa*, and the antibiotic was escalated from ceftriaxone to meropenem. Hospital course on day three was complicated by septic shock, worsening elevation of creatine kinase, and renal function. The patient was transferred to our facility for a higher level of care.

**Table 1:** Pertinent lab results on admission

Lab	Lab Value	Normal Range
White blood cells (WBCs)	25,800	4500-11,000/mm^3^
Lactic acid	3.3	0.5-2.2 mmol/L
Creatinine	4.1	0.6-1.2 mg/dL
Blood urea nitrogen (BUN)	52	7-18 mg/dL
Creatine kinase	680	25-90 U/L

**Figure 1:**
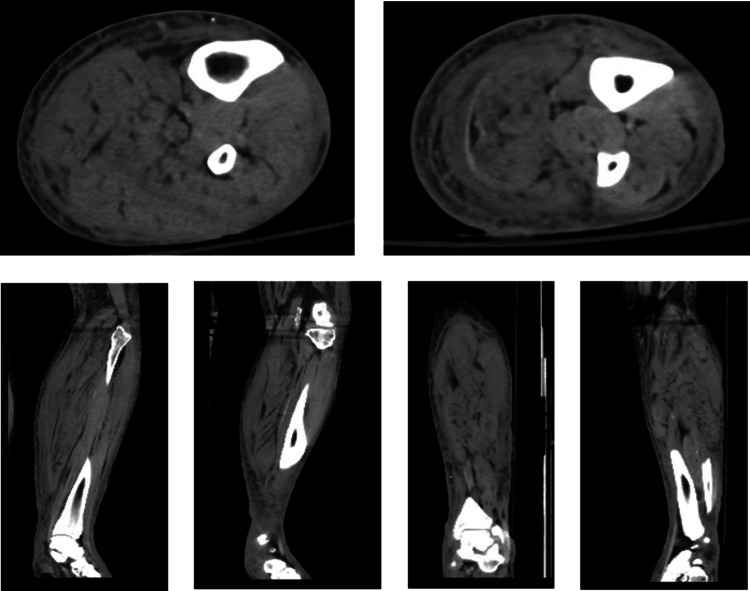
Soft tissue CT images of the coronal, sagittal, and frontal planes of the left lower extremity, confirming edema and subsequent cellulitis

On presentation, the examination showed tense left lower extremity swelling, multiple blisters on the anteromedial aspect of the lower portion, gray-colored skin in the distal leg, erythema in the thigh to the groin, severe pain that was out of proportion to the examination, and the presence of crepitus. The CT with contrast of the lower extremity showed soft tissue swelling suggestive of cellulitis (Figure 1). Due to concern for NF, the patient was emergently taken to the operating room. The patient underwent above-knee amputation (AKA) due to extensive necrosis and inflammation of the tissue. After the AKA procedure, the wound culture was found to grow *Pseudomonas aeruginosa*, which showed susceptibility to cefepime, gentamicin, levofloxacin, zosyn, ciprofloxacin, and meropenem. On day 3, the patient was taken back to the operating room for further debridement and placement of a wound vac.

The patient required continual hemodialysis and ventilator support. Initially post-operation, the patient was on cefepime with flagyl, but had developed progressive cellulitis, and was therefore switched back to meropenem and flagyl as per infectious disease recommendation. On day 10 post amputation, the patient was extubated and transferred to another facility for advanced plastic surgical closure of the wound in stable condition.

## Discussion

*Pseudomonas aeruginosa*-associated NF with sepsis is an exceptionally rare entity, with very few cases documented in the United States. It is most often seen in immunocompromised patients, though it remains uncommon. Untreated or poorly controlled CLL significantly compromises the immune system, thereby increasing susceptibility to infections. This occurs as a result of multifactorial dysfunction affecting both innate and adaptive immunity, accompanied by progressive hypogammaglobulinemia [5]. Our patient had a history of CLL and chronic kidney disease (CKD), both of which contributed to an immunocompromised state. In addition, advanced age and alcohol use further elevated his risk for NF due to impaired immune function, compromised tissue integrity, and increased exposure to pathogens [6]. A nationwide cohort study found that patients with alcohol use disorder had a 7.73-fold higher incidence of NF [6]. Laboratory findings demonstrated persistently elevated lymphocyte counts (55-87%) with smudge cells and neutropenia, consistent with CLL-related immunosuppression [7], which likely played a critical role in this presentation.

A reported case of *Pseudomonas*-induced NF was previously reported in an 85-year-old male with similar immunosuppression [4]. His past medical history was significant for early-stage gastric cancer and MALT (mucosa-assisted lymphoid tissue) lymphoma [4]. However, clinical presentations varied between our patients, as this patient presented with leukocytopenia and a negative creatine kinase at the time of admission. Clinicians empirically treated this patient with broad-spectrum antibiotics, including meropenem, but the patient was ultimately deemed a poor surgical candidate due to widespread progression of the disease. Another case reported *Pseudomonas* NF in a patient with a history of myeloma one year prior to admission [8]. This patient presented similarly to our patient, with arterial blood gases (ABGs) indicative of metabolic acidosis, markedly elevated creatine kinase, and no early indication of crepitus. Ultimately, the hospital course was significant for empiric antibiotic therapy with Zosyn and linezolid with no improvement, and unfortunately, the patient succumbed to multiorgan systemic failure [8]. In both reported cases of *Pseudomonas* NF, the patients had a history of immunosuppression and malignancy prior to admission. This may suggest a potential association between malignancy and this rare presentation of NF.

In one study comparing NF in patients with and without hematologic malignancies, infection was markedly more common in those with malignancies (87.5% vs. 9.5%) [9,10]. Another study showed that immunocompromised patients frequently lack the classic clinical features of NF, leading to significant diagnostic delays and worse outcomes [11].

Prompt diagnosis and treatment are essential to survival. Delays in diagnosis are strongly associated with increased mortality, emphasizing the need for a high index of suspicion. Early surgical intervention is particularly critical: surgery within 24 hours has been shown to significantly reduce mortality, whereas delays beyond 24 hours increase mortality from 35% to 53%, and failure to operate within three days results in 100% mortality [12]. The completeness of surgical debridement is also a major determinant of outcome, with radical debridement or incision and drainage techniques yielding the best survival even in patients with septic shock or multi-organ dysfunction syndrome [12]. Earlier intervention is necessary for these patients, as nonviable tissue can lead to tissue hypoxia, which can further limit the efficacy of intravenous antibiotics, disseminated intravascular coagulation, rhabdomyolysis, and compartment syndrome, as seen in our case.

Interestingly, a study of NF in patients with hematologic malignancies reported that delayed surgery did not worsen mortality compared to immunocompetent patients with gram-negative NF, with survival reaching 78% in the malignancy group [11]. This suggests that host factors, particularly immune status, may influence prognosis differently in this population, although the study attributed the findings to prompt recognition and diagnosis of NF in their center [8].

NF remains primarily a clinical diagnosis, as imaging may show nonspecific or even negative findings in the early stages. Nonetheless, imaging can be valuable to assess the extent of disease and assist surgical planning [13]. Importantly, treatment should not be delayed for imaging, as definitive diagnosis and management rely on surgical exploration, fasciotomy, aggressive debridement, and, in severe cases, amputation.

For patients presenting with suspected severe soft tissue infection and fever, empiric broad-spectrum antibiotics should be started immediately. If the infection progresses despite appropriate empiric therapy, clinicians must maintain a high suspicion for NF. Early blood cultures are vital, as pathogen identification and antibiotic susceptibility testing guide targeted therapy and improve outcomes [2]. Infectious Diseases Society of America (IDSA) guidelines were revised in 2014 to include proper management of NF [14].

## Conclusions

*Pseudomonas aeruginosa*-associated necrotizing fasciitis is an uncommon clinical entity, most often reported in immunocompromised patients. A high index of suspicion is essential, as early recognition and timely management can significantly reduce mortality in this vulnerable population. Optimal outcomes are best achieved through a multidisciplinary approach that combines prompt empiric antibiotics, rapid organism identification, and urgent surgical intervention. In this case, early surgical management was critical and contributed to the patient’s stabilization and recovery. There is one limitation in this case, as an anaerobic culture was not obtained at the time of presentation, which could exclude other bacteria involved in the presentation.

Our proposed interventions include creating specific guidelines for the early diagnosis of necrotizing fasciitis to preserve survival, incorporating timely blood cultures and guidelines for antibiotic treatment, early surgical intervention, and continual research into less common presentations of NF.
